# Proteomics of High-Grade Serous Ovarian Cancer Models Identifies Cancer-Associated Fibroblast Markers Associated with Clinical Outcomes

**DOI:** 10.3390/biom13010075

**Published:** 2022-12-30

**Authors:** Meinusha Govindarajan, Vladimir Ignatchenko, Laurie Ailles, Thomas Kislinger

**Affiliations:** 1 Department of Medical Biophysics, University of Toronto, Toronto, ON M5G 1L7, Canada; 2 Princess Margaret Cancer Center, University Health Network, Toronto, ON M5G 1L7, Canada

**Keywords:** high-grade serous ovarian cancer, cancer-associated fibroblast, mass spectrometry, proteomics, *N*-glycoproteomics, tumor microenvironment

## Abstract

The tumor microenvironment has recently emerged as a critical component of high-grade serous ovarian cancer (HGSC) disease progression. Specifically, cancer-associated fibroblasts (CAFs) have been recognized as key players in various pro-oncogenic processes. Here, we use mass-spectrometry (MS) to characterize the proteomes of HGSC patient-derived CAFs and compare them to those of the epithelial component of HGSC to gain a deeper understanding into their tumor-promoting phenotype. We integrate our data with primary tissue data to define a proteomic signature of HGSC CAFs and uncover multiple novel CAF proteins that are prognostic in an independent HGSC patient cohort. Our data represent the first MS-based global proteomic characterization of CAFs in HGSC and further highlights the clinical significance of HGSC CAFs.

## 1. Introduction

Epithelial ovarian cancer (EOC) is the most lethal gynecological malignancy in the USA, Canada, and Europe [1,2,3]. High-grade serous carcinoma (HGSC) is the most prevalent and aggressive subtype of EOC. Driven by high rates of cancer recurrence and subsequent chemoresistance, the low five-year survival rate for HGSC (~30%) [4] underscores the need for the development of novel therapies. Although most HGSC research efforts are devoted to investigating the epithelial cancer compartment [5], there is increasing recognition of the tumor microenvironment’s imperative role in HGSC disease progression, thus highlighting its value as a source of potential therapeutic targets and biomarkers. Cancer-associated fibroblasts (CAFs) are an abundant cell type in the tumor microenvironment and are characterized by their highly secretory phenotype [6]. In HGSC, CAFs have been shown to promote cancer cell proliferation [7], enable tumor dissemination through the abdominal cavity [8], facilitate immune suppression [9], and mediate chemotherapy resistance [10]. Hence, molecular characterizations of HGSC CAFs can contribute to a deeper understanding of this unmistakably important cell type and may help inform additional therapeutic strategies for HGSC.

Most efforts to gain molecular insights into HGSC CAF biology have been focused on the transcriptome [11,12], but mRNA expression is a poor surrogate for protein expression, especially for secreted and cell surface proteins [13] and the global proteomes of HGSC CAFs have yet to be directly investigated. Here, we use mass spectrometry (MS) to characterize the proteomes of HGSC patient-derived CAFs and compare them to those of HGSC epithelial and normal epithelial cell lines of the fallopian tube. We show that in vitro proteomic trends concur with the proteomes of primary tissue samples, supporting the utility of these models. Finally, we integrated our in vitro data with HGSC tissue proteomics data to determine a proteomic signature of HGSC CAFs and identify multiple previously undescribed CAF proteins with prognostic significance in an independent HGSC patient cohort. Our work provides novel proteomic insights regarding HGSC CAFs and points towards additional evidence accentuating the importance of the tumor microenvironment in HGSC.

## 2. Materials and Methods

### 2.1. Cell Lines and Culture Conditions

HGSC patient-derived CAFs were isolated as previously described [11] and generously provided by the Ailles lab. Commercially available HGSC cell lines were graciously provided by the Hakem lab and authenticated using Short Term Repeat (STR) DNA profiling at TCAG Facilities (The Hospital for Sick Children, Toronto, ON, Canada). Immortalized FTE cells were generously provided by the Rottapel lab. Primary CAFs and immortalized epithelial cells were cultured in IMDM or RPMI, respectively, supplemented with 10% fetal bovine serum (FBS) and p enicillin-streptomycin-glutamine (PSG) (100 U/mL penicillin, 100 μg/mL streptomycin, 292 μg/mL L-glutamine, Gibco). Primary CAFs and immortalized epithelial cells were incubated at 37 °C in 5% CO_2_/2% O_2_ and 21% O_2_, respectively.

### 2.2. Cell Lysis and Protein Digestion

Each cell line was processed in four individual replicates per sample preparation method (i.e., for each cell line, cells were collected from four different cell culture plates and processed in four separate sample preparations). Cell pellets were resuspended in 50% (*v/v*) 2,2,2-trifluouroeothanol (TFE) in phosphate-buffered saline and lysis was accomplished with five freeze–thaw cycles and pulse sonication followed by a two-hour incubation at 60°C with agitation every 30 min. Protein concentration was determined using the BCA assay (Pierce) according to the manufacturer’s instructions. 100 µg and 1 mg of protein lysate were used for whole cell lysate (WCL) and *N*-glycocapture (GLYCO) samples, respectively. A total of 2 pmol of yeast invertase (SUC2) was spiked into each sample as an internal control. Cysteines were reduced with 5 mM dithiothreitol at 60 °C for 30 min and subsequently alkylated using 25 mM iodoacetamide at room temperature in the dark for 30 min. Lysates were diluted 1:5 (*v/v*) with 100 mM ammonium bicarbonate (pH 8.0) with 2 mM calcium chloride. Mass-spectrometry grade Trypsin-LysC (Promega) was added to the samples at a ratio of 1:100 (enzyme:protein lysate) and digestion was performed overnight at 37 °C. The digestion was quenched with 0.5% formic acid. Tryptic peptides for whole cell lysate (WCL) and *N*-glycocapture (GLYCO) fractions were desalted using C18 stage tips (3M Empore™) or MacroSpin columns (Nest group), respectively, and lyophilized using a vacuum concentrator.

### 2.3. N-glycopeptide Enrichment

Enrichment of *N*-glycopeptides was performed as previously described [14,15]. Briefly, lyophilized peptides were resuspended in a coupling buffer (100 mM sodium acetate, 150 mM sodium chloride, pH 5.5) and glycans were oxidized using 10 mM sodium metaperiodate at room temperature for 30 min in the dark. The excess sodium metaperiodate was removed via C18 desalting and followed by lyophilization. Peptides were resuspended in coupling buffer and oxidized glycopeptides were coupled to hydrazide magnetic beads (Chemicell) overnight with constant rotation. The supernatant containing non-glycosylated peptides was discarded and the beads bound with *N*-glycopeptides were washed (5× each) with coupling buffer, 1.5 M NaCl, water, methanol, 80% acetonitrile and 100 mM ammonium bicarbonate to remove non-specific peptides. *N*-glycopeptides were enzymatically de-glycosylated and eluted from the beads with 5 units of PNGase F (Roche) in 100 mM ammonium bicarbonate at 37 °C overnight. The de-glycosylated peptides were subsequently desalted using C18 stage tips and lyophilized.

### 2.4. Mass Spectrometry-Based Proteomics

Peptides were resuspended in 0.1% formic acid in LC-MS grade water and concentration was determined using a Nanodrop 2000 (Thermo Scientific, Waltham, MA, USA). Using an EASY nLC1000 nano-flow liquid chromatography system (Thermo Scientific), 1.5µg of peptides were loaded onto a 50 cm EasySpray ES803 column (Thermo Scientific) coupled to an Orbitrap QExactive Mass Spectrometer (Thermo Scientific). WCL peptides were separated using a four-hour chromatographic gradient and GLYCO peptides were separated with a two-hour gradient. Mass spectrometry data were acquired in data-dependent, top-20 mode. For WCL samples, MS1 spectra were acquired for a mass range of 350–1550 *m/z* at a resolution of 140,000 at 200 *m/z* (FWHM), with an automatic gain control (AGC) target of 3 × 10^6^ and a maximum ion fill time of 220 ms. The MS2 profiles were acquired at a resolution of 17,500 at 200 *m/z* (FWHM), with an AGC target of 5 × 10^5^ and maximum ion fill time of 25 ms. The isolation width was set at 2 *m/z* and isolation offset at 0.4 *m/z*. A normalized collision energy of 27% was used and dynamic exclusion was set to 40 s. For GLYCO samples, MS1 spectra were acquired for a mass range of 350–1800 *m/z* at a resolution of 70,000 at 200 *m/z* (FWHM), with an AGC target of 3 × 10^6^ and maximum ion fill time of 240 ms. MS2 profiles were acquired at a resolution of 17,500 at 200 *m/z* (FWHM), with an AGC target of 2 × 10^5^ and a maximum fill time of 100 ms. The isolation width was set at 2 *m/z* and isolation offset at 0.4 *m/z*. A normalized collision energy of 27% was used and dynamic exclusion was set to 40 s. MaxQuant (version 1.6.3.3) [16] was used to search acquired data against a merged UniProt protein database comprised of human sequences and the yeast invertase (SUC2) sequence, with match-between-runs enabled. For each fraction, CAF samples were searched separately to minimize artificial matching from the epithelial sample runs and vice versa through the match-between-run function. In all searches, a maximum of two missed cleavages were allowed, carbamidomethylation of cysteine was specified as a fixed modification and oxidation of methionine was specified as a variable modification. Specifically for *N*-glycocapture searches, deamidation of asparagine to aspartic acid (as a result of the PNGase F elution) was also included as a variable modification. The false discovery of peptides was controlled using a target-decoy approach based on reversed sequences, and defined as 1% at site, peptide, and protein levels. For WCL samples, the peptides.txt MaxQuant output files were parsed into an in-house database for protein grouping and intensity-based absolute quantification (iBAQ) values were used for protein quantification. The MaxQuant output file: Asn-AspSites.txt was used for GLYCO data analysis. Peptides detected with an asparagine deamidation modification within the *N*-glycosylation *N*-[!*P*]-*STC* sequon (*N* = asparagine; [!*P*] = any amino acid other than proline; STC = serine, threonine, or cysteine at the +2 site) and with a localization probability > 0.8 were considered *N*-glycopeptides and were used in subsequent analyses. *N*-glycopeptide intensities were summed for *N*-glycoprotein quantification.

### 2.5. Differential Protein Expression Analysis

Data were median normalized and missing protein intensities were imputed with random values from a lower normal distribution (width = 0.2, down-shift = 1.8). A one-way ANOVA followed by multiple testing corrections using the Benjamini–Hochberg method was used to determine proteins that were differentially expressed between the three cell types. A cell type elevated protein was defined as a significantly differentially expressed protein that had a Tukey’s post hoc *p*-value < 0.05 and |log_2_fold change (FC)| > 1 for the cell type against each of the other two cell types. For example, a CAF elevated protein is a protein that has a log_2_FC (HGSC/CAF) < −1, Tukey’s *p*-value (HGSC vs CAF) < 0.05, log_2_FC (FTE/CAF) < −1, and Tukey’s *p*-value (FTE vs CAF) < 0.05. From the remaining differentially expressed proteins, a cell type shared protein was defined as a protein that had Tukey’s post hoc *p*-values < 0.05 and |log_2_FC| > 1 for two cell types against the remaining cell type. For example, a HGSC + FTE shared protein is a protein that has a log_2_FC (HGSC/CAF) > 1, Tukey’s *p*-value (HGSC vs CAF) < 0.05, log_2_FC (FTE/CAF) > 1 and Tukey’s *p*-value (FTE vs CAF) < 0.05.

### 2.6. Pathway Analysis

gProfiler [17] was used for all pathway analyses. For fraction comparison, over- and under-representation analysis was performed separately on each fraction and select Gene Ontology: Cellular Component terms that were statistically significant (adjusted *p*-value < 0.05) in each fraction were visualized. For WCL cell type elevated and shared comparisons, the top four non-redundant statistically significant (adjusted *p*-value < 0.05) Gene Ontology: Biological Processes terms were visualized for each cell-type annotation group. For both analyses, the whole human proteome was used as a background.

### 2.7. Comparison to Tissue Proteomics Datasets

Differential expression analysis annotations (e.g., epithelial or stromal enriched), tumor cellularity values, and proteomic subtype classification annotations were obtained from the supplemental tables of the respective published tissue datasets [18,19,20]. To evaluate the enrichment of cell-type signatures in the CPTAC cohort, we used gene set variation analysis (GSVA), which is a method of gene set enrichment used for individual samples, to calculate sample-wise enrichment scores [21].

### 2.8. Survival Analysis

Proteomic and clinical outcome data from the CPTAC cohort of 169 HGSC patients [20] was used to determine associations between the expression of high-confidence CAF and HGSC proteins and overall survival/recurrence-free survival. For each protein, patients were median dichotomized based on protein expression and hazard ratios and confidence intervals were calculated using a univariate Cox-proportional hazards model (survival package in R). Proteins in each signature (i.e., CAF and HGSC) were tested separately, followed by multiple hypothesis correction of *p*-values from the log-rank test using the Benjamini–Hochberg method.

## 3. Results

### 3.1. Proteomic Profiling of In Vitro HGSC Models

**Figure 1:**
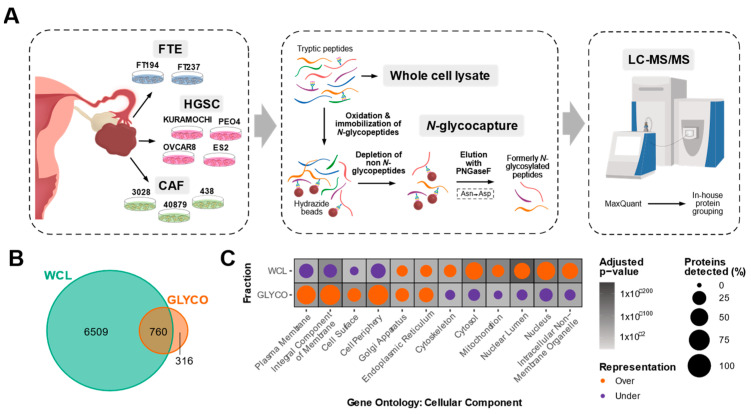
**Overview of in vitro HGSC proteomic characterization**. (**A**) Schematic of in vitro proteomic workflow. Whole cell lysate (WCL) and *N*-glycopeptide (GLYCO) enriched fractions of three high-grade serous ovarian cancer (HGSC) patient-derived cancer-associated fibroblast (CAF) cultures, four immortalized HGSC epithelial lines and two immortalized normal fallopian tube epithelial (FTE) lines were analyzed by liquid chromatography-tandem mass spectrometry (LC-MS/MS). Each cell line was processed in four replicates. (**B**) Venn diagram of proteins detected in each fraction. (**C**) Dot plot depicting statistically significant over- (orange) and under-represented (purple) Gene Ontology: Cellular Component terms in each fraction. Size of the circles correspond to the percentage of proteins detected in each fraction annotated with the respective term and the background shading indicates the adjusted p-value.

Despite accumulating evidence shedding light on the clinical importance of CAFs in HGSC, proteomic characterizations of HGSC CAFs remain limited. To profile the proteome of HGSC CAFs and compare it to that of the HGSC epithelial compartment (cancer and normal cells), we performed liquid chromatography-tandem mass spectrometry (LC-MS/MS) based proteomics on whole cell lysate (WCL) from three patient-derived cancer-associated fibroblast lines (CAF3028, CAF40879, CAF438), four HGSC cancer cell lines (KURAMOCHI, OVCAR8, ES2, PEO4), and two immortalized fallopian tube secretory epithelial cell lines (FT194, FT237) (Figure 1A). The HGSC cell lines were selected due to their close resemblance to the molecular profiles of HGSC tumors [22] and FTE cells have recently been identified to be the cell of origin for HGSC [23,24], hence serving as a non-cancerous epithelial control. CAFs are defined by a highly secretory phenotype and as most secreted proteins are predicted to be *N*-glycosylated [25], we rationalized that *N*-glycoproteomics can complement a WCL proteomics analysis to gain deeper insights into CAF biology. Hence, we also used an established *N*-glycopeptide-based enrichment strategy (Figure 1A) to profile the *N*-glycoproteome of 8/9 in vitro models. In total, we detected 7585 proteins across both fractions (Table S1). A total of 316 proteins were uniquely detected in the *N*-glycoproteome (GLYCO) dataset (Figure 1B), highlighting how *N*-glycoproteomics can provide additional proteomic information that may be neglected in a WCL analysis. The high correlation between cell line replicates in WCL (median pairwise cell line replicate Pearson’s *r* = 0.95, Figure S1A,B) and GLYCO (median pairwise cell line replicate Pearson’s *r* = 0.92, Figure S1C,D) fractions and minimal variability of an internal control spike-in protein (yeast invertase-SUC2) across runs (Figure S1E) confirmed high data reproducibility. While the WCL proteome was significantly over-represented in cytosolic proteins and significantly under-represented in surface-localized and extracellular-localized proteins, the opposite trend was observed in the GLYCO fraction (Figure 1C). This comparison illustrates the bias of WCL proteomics analyses towards the detection of intracellular proteins and the value of *N*-glycoproteomics for extracellular protein enrichment.

**Figure 2:**
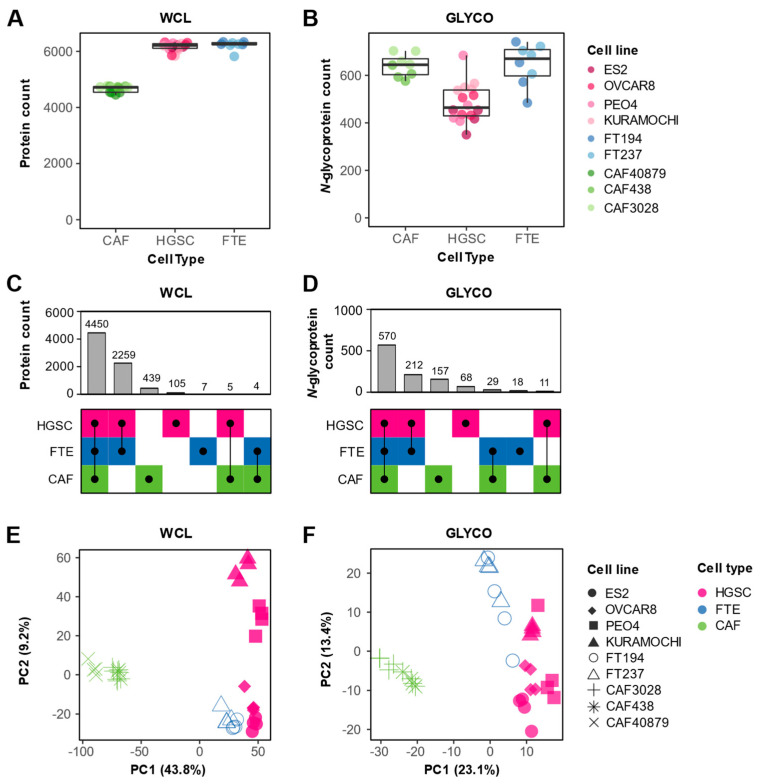
**Proteomic cell type heterogeneity.** (**A**,**B**) Box plots showing number of proteins detected by cell type in (**A**) WCL and (**B**) GLYCO fractions. Dots represent number of proteins detected in each processing replicate (*n* = 4) and are colored based on cell line. (**C**,**D**) UpSet plots depicting protein detection differences between CAFs, HGSC, and FTE cells in (**C**) WCL and (**D**) GLYCO fractions. (**E**,**F**) Principal component analysis of (**E**) WCL and (**F**) GLYCO proteomic data. Each point represents a cell line-processing replicate (*n* = 4), and the shape indicates respective cell line. Color is used to denote cell type.

We observed differences in protein detection between cell types. In the WCL fraction, we detected the least number of proteins in CAFs (median of 4718, 6214, and 6257 proteins per CAF, HGSC, and FTE replicate, respectively) (Figure 2A). In the GLYCO fraction, CAFs and FTE cells contained the most proteins (median of 645, 464 and 670 glycoproteins per CAF, HGSC, and FTE replicate, respectively) (Figure 2B). Interestingly, we also observed that glycoproteins are of a higher abundance in CAFs (Figure S2A). This is in contrary to the lower abundance of glycoproteins in HGSC cells and a lack of statistically significant difference in glycoprotein abundance in FTE cells (Figure S2A), suggesting that *N*-glycoproteins are differentially regulated in CAFs. Qualitatively, there was a higher overlap of protein (Figure 2C) and N-glycoprotein (Figure 2D) detection between HGSC and FTE cells compared to CAFs. This is expected as both HGSC and FTE cells are of a shared epithelial lineage whereas CAFs are a mesenchymal cell type. Indeed, several epithelial and/or HGSC markers (e.g., EPCAM, MUC16, PAX8) were exclusively detected in the HGSC and FTE cells (Figure S2B). Principal component analysis (PCA) of both WCL (Figure 2E) and GLYCO (Figure 2F) data demonstrated that the cell lines clustered based on cell type. For both fractions, the first principal component can be explained by the difference between the CAFs and the two epithelial cell types, further supporting the large proteomic differences between mesenchymal and epithelial cellular programs. Despite the qualitative similarities in protein detection between HGSC and FTE cells, PCA also revealed that the in vitro cancer and normal epithelial proteomes differ quantitively.

### 3.2. Characterization of Cell Type Elevated and Shared Proteins

**Figure 3:**
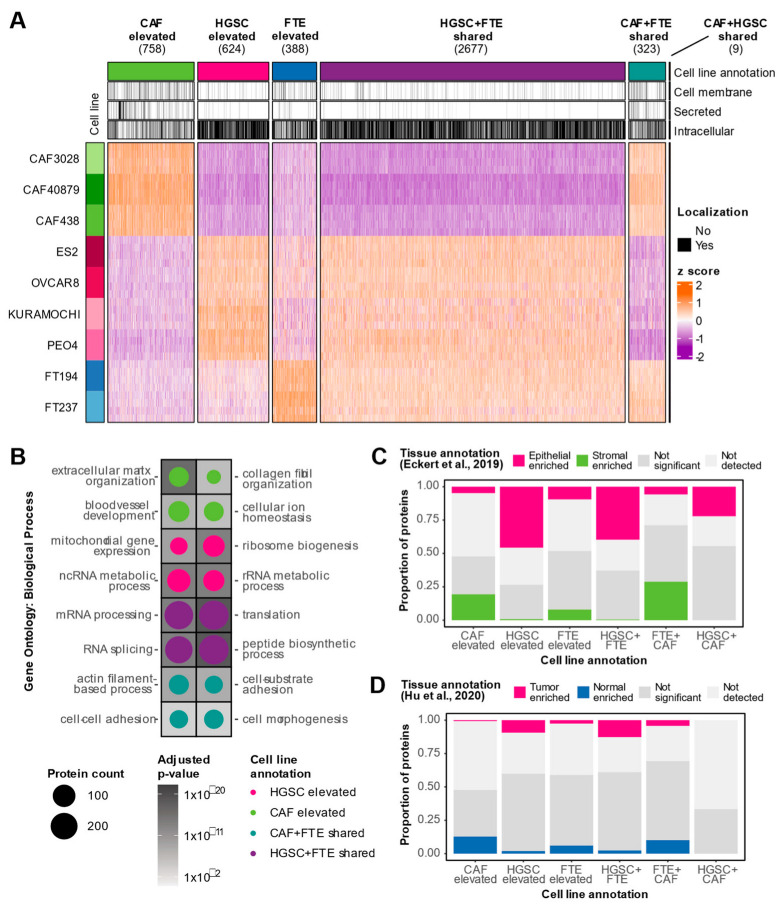
**Characterization of in vitro cell type elevated and shared proteins.** (**A**) Heatmap visualizing protein expression of cell type elevated (Tukey’s *p*-value < 0.05 and |log_2_FC| > 1 against two cell types) and shared (Tukey’s *p*-value < 0.05 and |log_2_FC| > 1 against one cell type) proteins detected in WCL. Covariate bars indicate UniProt subcellular localization keyword annotations. (**B**) Significantly over-represented Gene Ontology: Biological Processes in cell type elevated and shared proteins. Size of the circle represents the number of proteins detected in each cell type classification annotated with the respective term and the shading of the background tile indicates the adjusted p-value. No pathways passed statistical significance for FTE elevated and CAF + HGSC shared cell type annotations. (**C**) Stacked bar plot depicting proportion of cancer epithelial and cancer stromal enriched tissue proteins [18] in each WCL cell type classification group. (**D**) Stacked bar plot depicting proportion of tumor and normal enriched tissue proteins [19] in each WCL cell type classification group.

To determine proteins that underlaid the observed cell type heterogeneity, a one-way ANOVA was performed on the WCL data, and 6009 proteins were found to be differentially expressed between the three cell types at an FDR of 5%. A |log_2_FC|> 1 and Tukey’s *p*-value < 0.05 threshold was applied to determine cell-type elevated (i.e., proteins with higher expression in one cell type compared to the other two) and cell type shared (i.e., proteins with higher expression in two cell types compared to the other cell type) proteins (Figure 3A). This comparative analysis identified 758 CAF-elevated proteins, 624 HGSC-elevated proteins, and 388 FTE-elevated proteins. CAF-elevated proteins were over-represented in pathways known to be associated with CAFs such as extracellular functions and collagen organization (Figure 3B, Table S2). HGSC-elevated proteins were conversely enriched in processes consistent with cell proliferation such as RNA and mitochondrial functions (Figure 3B, Table S2). As anticipated by the PCA separation, HGSC and FTE cells share a core set of proteins (2677) that were not highly expressed by CAFs. Interestingly, 323 proteins were deemed to be CAF + FTE shared proteins. These CAF + FTE shared proteins comprised a higher proportion of secreted proteins (Figure 3A), suggesting that these proteins may represent minutiae associated with the secretory phenotype of both cell types. Overall, the biological processes underlying the in vitro cell type heterogeneity recapitulates known pathways of these cell types.

### 3.3. In Vitro Proteomics Reflects Tissue Proteomic Profiles

We next evaluated how in vitro proteomic trends related to tissue proteomic profiles. First, we compared our WCL cell type annotations to proteins that were identified as differentially expressed between HGSC stroma and HGSC epithelium in a laser-capture microdissection (LCM) proteomics study of 11 HGSC patients [18]. CAF-elevated proteins were enriched in cancerous stromal-associated tissue proteins while HGSC elevated cell line proteins were enriched in cancerous epithelial-associated tissue proteins, demonstrating agreement between in vitro models and tissue proteomes (Figure 3C). We also noted that HGSC + FTE shared proteins were enriched in cancerous epithelial-associated tissue proteins, implying that this set of epithelial-associated proteins may not be cancer-specific. Similarly, CAF + FTE shared cell line proteins were enriched in cancer stromal-associated tissue proteins, suggesting that this subset of stromal-associated proteins may be proteins associated with a general secretory phenotype rather than a specific cancer phenotype. This highlights how cell type-specific information may be obscured in tissue proteomics data. We also compared our WCL cell-type elevated and shared proteins to data generated by Hu et al., [19] where the bulk proteomes of 80 HGSC tumors and 20 normal fallopian tubes were characterized. HGSC cell line-elevated proteins were enriched in cancer-associated tissue proteins and FTE cell line-elevated proteins comprised a higher proportion of normal associated tissue proteins (Figure 3D). In sum, these data suggest that in vitro WCL proteomic trends concur with HGSC WCL tissue proteomes.

### 3.4. Integration of In Vitro and Tissue Proteomics Data Reveals CAF Enriched Proteins Associated with Clinical Outcomes

**Figure 4:**
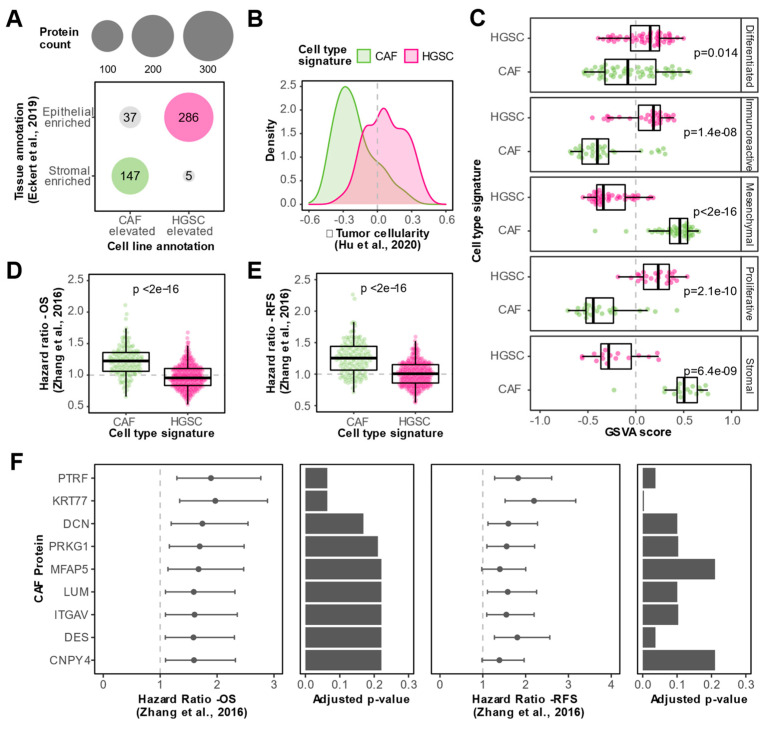
**Identification of CAF proteins associated with clinical outcomes in HGSC.** (**A**) Dot plot visualizing the integration between WCL cell line and tissue data [18] for the identification of cell type signatures. Proteins with cell line annotations that coincide with tissue annotations are colored and denoted as high-confidence CAF (green) or HGSC (magenta) proteins. Size of circles correspond to the number of proteins. (**B**) Density plot showing spearman correlations with tumor cellularity in Hu et al., 2020 dataset [19] for proteins in the CAF (green) or HGSC (magenta) signatures. (**C**) Box plots illustrating gene set variation analysis (GSVA) scores for high-confidence CAF (green) and HGSC (magenta) proteins in HGSC tumor proteomes, stratified by HGSC proteomic subtypes determined by Zhang et al. [20]. Dots indicate GSVA scores of individual HGSC tumor samples. *p*-values from a Student’s *t*-test between CAF and HGSC GSVA scores are reported. (**D**) Box plots comparing overall survival (OS) hazard ratios of proteins in CAF and HGSC signatures based on data from Zhang et al., [20]. Dots represent hazard ratios of individual proteins in each signature. *p*-value from a student’s *t*-test is reported. (**E**) Box plots comparing recurrence-free survival (RFS) hazard ratios of proteins in CAF and HGSC signatures based on data from the Zhang et al., 2016 dataset [20]. Dots represent hazard ratios of individual proteins in each signature. *p*-value from a student’s *t*-test is reported. (**F**) Hazard ratios (HR) and confidence intervals for nine CAF-enriched proteins that were statistically significantly associated with both poor overall survival and poor recurrence-free survival (FDR < 0.25 and HR > 1) in Zhang et al. 2016 cohort [20]. Bar plots show the Benjamini–Hochberg adjusted *p*-value from the log-rank test for the respective clinical outcome.

Since tissue datasets are susceptible to masking cell type heterogeneity, we reasoned that the integration of our in vitro proteomic data with published tissue datasets will help uncover genuine CAF-associated markers. We considered the intersection of CAF elevated in vitro proteins and stromal-enriched tissue proteins as high-confidence CAF proteins. Similarly, we classified the intersection of HGSC elevated in vitro proteins and epithelial enriched tissue proteins as high-confidence HGSC proteins. This approach led to the identification of a CAF signature comprising 147 high-confidence CAF-derived proteins and a HGSC signature consisting of 286 high-confidence HGSC-derived proteins (Figure 4A). In a large proteomic cohort of 80 HGSC bulk tumor tissues [19], the set of high-confidence CAF proteins was negatively associated with tumor cellularity whereas the high-confidence HGSC proteins tended to be positively associated with tumor cellularity (Figure 4B), independently supporting the aptness of our cell type signatures.

To assess the clinical relevance of CAF proteins in HGSC, we leveraged proteomic and clinical outcome data from an independent cohort of 169 HGSC tumors profiled by the Clinical Proteomic Tumor Analysis Consortium (CPTAC) [20], the largest HGSC tumor cohort profiled by MS to date. We used our cell type signatures to show that the mesenchymal and stromal HGSC subtypes identified by CPTAC are enriched in the CAF signature, whereas the other three subtypes were enriched in the HGSC cell type signature (Figure 4C). This proteomic data is in-line with transcriptomic data that suggests that the mesenchymal HGSC subtype is driven by CAFs rather than cancer epithelial cells merely undergoing epithelial to mesenchymal transition [26], further supporting the reliability of our cell type signatures. Interestingly, we found that proteins in our CAF signature were associated with poorer overall survival (Figure 4D) and recurrence-free survival (Figure 4E) compared to HGSC proteins (Table S3). Out of the 147 high-confidence CAF proteins, 10 proteins were negatively associated with overall survival and 49 proteins were negatively associated with recurrence-free survival (HR > 1 and FDR < 25%) in the CPTAC cohort. The nine CAF proteins that were negatively associated with both overall survival and recurrence-free survival are visualized in Figure 4F (HR > 1 and FDR < 25%). These prognostic CAF proteins include previously described HGSC CAF markers (e.g., MFAP5 and LUM) and novel HGSC CAF proteins such as CNPY4, KRT77, and PTRF. Conversely, no HGSC proteins were negatively associated with overall survival and/or recurrence-free survival (HR > 1 and FDR < 25%) in the CPTAC cohort. Together, these results highlight the clinical significance of CAFs in HGSC.

### 3.5. N-glycoproteomics Enables Identification of Additional Prognostic CAF Proteins in HGSC

Provided the enrichment of *N*-glycoproteins in CAFs, we lastly investigated whether *N*-glycoproteomic analysis can offer further proteomic insights into CAFs. We used the same approach as in the WCL analyses to determine cell-type elevated and shared *N*-glycoproteins and observed that there was an overall agreement of the in vitro classifications between the WCL and GLYCO analyses (Figure S3A). The 205 CAF-elevated *N*-glycoproteins identified in the GLYCO analysis were uniquely enriched in specific biological process terms related to angiogenesis and wound healing compared to the WCL CAF-elevated proteins (Figure S3B). Despite the large overlap, the GLYCO analysis identified 55 CAF-elevated proteins that were not detected in the WCL in vitro experiments. As many of these GLYCO unique, CAF-elevated proteins were also not detected in the WCL LCM study by Eckert et al., (Figure S3C), it is likely that these GLYCO unique proteins may be lower abundance proteins that are neglected in WCL analyses. Nevertheless, we were able to validate tissue stromal enrichment for five GLYCO unique, CAF-elevated proteins (APOD, ENTPD1, EMILIN3, MFAP4, and OLFML3). Interestingly, OLFML3 was significantly associated with poor overall survival and EMILIN3, ENTPD1, and OLFML3 were significantly associated with poor recurrence-free survival in the HGSC CPTAC cohort (HR > 1 and FDR < 25%) (Figure S3D). These data indicate that *N*-glycopeptide-based enrichment methods can help uncover clinically relevant proteins which otherwise may have been ignored by a classical WCL proteomics approach.

## 4. Discussion

The tumor microenvironment, comprised of CAFs, immune cells, vascular endothelial cells, and ECM, is a permissive niche that facilitates HGSC progression [27]. CAFs represent a critical cellular component of the tumor stroma as they engage in bi-directional communication with cancer cells to directly promote cancer cell proliferation, invasion, and metastasis [28]. CAFs also interact with other components of the tumor stroma as they secrete factors to remodel the ECM, promote angiogenesis, and assist in immune evasion [6]. A higher abundance of CAFs has been shown to be associated with advanced-stage disease and increased omental metastases in ovarian cancer [29], further highlighting their importance in ovarian cancer pathogenesis. Molecular characterizations of CAFs in HGSC are needed to gain a deeper understanding of features underlying the pro-tumorigenic role of CAFs. Although transcriptomic profiling of HGSC CAFs has provided novel insights such as the identification of multiple CAF states and new markers of the CAF state [11,12,26], mRNA abundance only weakly correlates with protein abundance [13] and transcriptomics cannot provide information regarding post-translational modifications (PTMs). While Curtis et al. [30] have previously characterized the phosphoproteome of CAFs in HGSC, global proteomic profiling of HGSC CAFs has yet to be conducted. To address this gap, here we have used MS to characterize the proteomes of HGSC patient-derived CAFs and compare them to those of HGSC and fallopian tube epithelial cells.

Due to their ease of use and accessibility, in vitro models are valuable preclinical tools to investigate cancer biology. It is, however, imperative that experimental models accurately replicate disease biology to warrant findings with maximum translational utility. HGSC research has previously mistakenly fallen victim to the use of poor experimental models. Domcke et al., [22] compared the molecular profiles of 47 EOC cell lines and 316 HGSC tumor samples and concluded that the most used epithelial cancer cell lines in HGSC research poorly agreed with the molecular features of HGSC. A proteomic profiling study of 28 EOC cell lines revealed distinct clusters of cell lines likely due to differences in the tissue of origin [31]. Together these studies underscore the value of molecularly characterizing experimental models to facilitate physiologically relevant research. Here, we show that our models express known cell type markers, and that the proteomes are reflective of underlying biological processes characteristic of these cell types. We also compared in vitro WCL proteomes to HGSC tissue WCL proteomes [18,19] and show overall concordance in directionality between stromal, cancer, and normal protein expression. There are some minor discrepancies which is expected as our in vitro proteomic data is of individual monoculture cell types and tissue proteomics datasets are a mixture of protein information derived from multiple cell types (e.g., tumor stroma is comprised of CAFs, immune cells, vasculature, and ECM [27]). Moreover, the enrichment of tumor and normal tissue proteins was of a lower magnitude compared to the enrichment of cancer epithelial and cancer stromal tissue proteins, likely due to increased cell type heterogeneity in bulk tissue samples compared to LCM tissue samples. Nevertheless, the overall concurring trends between in vitro and tissue proteomes suggest that our in vitro models represent suitable models to investigate HGSC biology.

Next, we integrated our in vitro WCL proteomics data with data from the LCM WCL tissue proteomic study of HGSC epithelium and stroma [18] to identify proteomic signatures of CAFs and HGSC cells, respectively. Though our approach of investigating individual cell types is advantageous for identifying cell-type-enriched proteomic features, a limitation of our work is that the artificial environment of in vitro culturing is likely to alter the proteome. Conversely, though tissue proteomics data will provide more physiologically relevant insights, as discussed above, it is difficult to delineate cell type information from such datasets alone. Hence, we rationalized that the integration of both data sources will leverage complementary benefits and enable the identification of bona fide CAF and HGSC proteins. Our proteomic signatures were comprised of 147 and 286 proteins enriched in CAFs and HGSC epithelial cells, respectively, though we cannot preclude expression in other cell types not investigated in our study. We used our signatures to show that CAF proteins are associated with poorer overall and recurrence-free survival compared to HGSC proteins in the CPTAC study of 169 HGSC patients [20]. 50 CAF proteins were significantly negatively associated with overall survival and/or recurrence-free survival while no HGSC proteins were significantly associated with poor overall survival and/or recurrence-free survival (HR > 1 and FDR < 25%). Nine proteins in the CAF signature were statistically significantly associated with both poor overall and recurrence-free survival (HR > 1 and FDR < 25%) in this cohort. Our analysis independently validated the prognostic utility of previously identified CAF-enriched proteins in EOC, such as MFAP5 [32] and LUM [33]. We also uncovered three CAF-enriched proteins (PTRF, CNPY4 and KRT77) that have not previously been implicated in the context of HGSC, let alone EOC, and may represent novel prognostic markers of HGSC. Stromal PTRF has been reported to be anti-tumorigenic and associated with better outcomes in prostate cancer [34,35], suggesting that the stromal role of this protein may be cancer-type specific. On the contrary, the functions of CNPY4 and KRT77 in CAF biology have yet to be investigated and future functional interrogations are required to elucidate the roles of these novel CAF-elevated proteins in HGSC tumorigenesis.

Finally, we used a *N*-glycopeptide-based enrichment method to complement the WCL proteomic analysis. *N*-glycosylation is a co-translational modification that is involved in the stability, solubility, and localization of proteins [36], and aberrant glycosylation has been recognized as a vital component of several hallmarks of cancer [37]. Since this covalent addition occurs in the canonical secretory pathways, most proteins destined for the cell surface (>80%) are predicted to be *N*-glycosylated [25]. Consequently, we hypothesized that a *N*-glycoproteomic analysis can provide additional proteomic insights into CAFs. Consistent with this, we observed that CAFs were enriched in *N*-glycoproteins compared to epithelial cells and that the *N*-glycoproteomic method enabled the identification of CAF-elevated proteins that were not detected in the in vitro WCL analysis. Compared to the CAF-elevated proteins identified in the in vitro WCL analysis, the CAF-elevated *N*-glycoproteome was uniquely enriched in biological process terms relating to wound healing and angiogenesis, suggesting that proteins involved in these CAF-associated processes are likely of a lower abundance and consequently, can be omitted in WCL proteomic analyses. The majority of GLYCO unique, CAF-elevated proteins were also not detected in the previously published WCL stromal dataset and therefore precluded our validation of tissue stromal expression. Despite this, our GLYCO analysis still identified three CAF-elevated proteins (OLFML3, ENTPD1, and EMILIN3) that were stromal enriched in HGSC tissue, associated with poor clinical outcomes in HGSC, and not detected in the in vitro WCL experiments, hence supporting the supplemental value of *N*-glycoproteomic analyses. Interestingly, OLFML3, ENTPD1, and members of the EMILIN family are reported to be involved in regulating angiogenesis in other cancers [38,39,40], which is in line with the unique enrichment of angiogenic processes in the CAF-elevated *N*-glycoproteome observed in our study. Our data warrant prospective stromal-focused *N*-glycoproteomic analyses in primary tissue to explore the full potential utility of CAF-enriched *N*-glycoproteins in HGSC.

In conclusion, we report the first global proteomic characterization of CAFs in HGSC. We show that in vitro proteomic trends align with tissue proteomes, supporting the reliability of these models. We also identify a proteomic signature of CAFs in HGSC and uncover multiple previously undescribed CAF proteins that are associated with poor clinical outcomes in HGSC. Avenues of future research include profiling additional HGSC CAF lines to define the proteomic features underlying CAF heterogeneity, and the proteomic characterization of co-culture models to elucidate bi-directional communication between cancer cells and CAFs.

## Data Availability

Raw mass spectrometry data are publicly available from UCSD’s MassIVE database (ftp://massive.ucsd.edu) with the following MassiVE ID: MSV000090794 and FTP link: ftp://massive.ucsd.edu/MSV000090794/. Processed proteomics data are available in Table S1.

## Supplementary Materials

The following supporting information can be downloaded at: https://www.mdpi.com/article/10.3390/biom13010075/s1, Figure S1: Quality control of proteomics data. (A) Pearson correlation plot of protein intensities of all WCL samples. (B) Box plot showing pairwise Pearson correlations between WCL cell line replicates (i.e., processing replicates, *n* = 4), cell-type replicates and all samples. Each dot represents a pairwise Pearson correlation. (C) Pearson correlation plot of *N*-glycoprotein intensities of all GLYCO samples. (D) Box plot showing pairwise Pearson correlations between GLYCO cell line replicates (i.e., processing replicates, *n* = 4), cell-type replicates and all samples. Each dot represents a pairwise Pearson correlation. (E) Box plot of SUC2 protein intensity across all samples. Dots indicate SUC2 protein intensities in individual samples; Figure S2: Additional proteomic cell type differences. (A) Log_2_ intensity distributions of all proteins detected in WCL (green) and proteins detected in both WCL and GLYCO fractions (brown) for each cell type. Each dot represents a protein intensity. *p*-values from a Student’s *t*-test are reported. (B) Heatmap showing mean log_2_ protein intensity of known CAF/mesenchymal markers (green) and HGSC/epithelial markers (magenta) in WCL samples of all cell lines. Gray indicates that the protein was not detected in the cell line; Figure S3: Characterization of the CAF-elevated *N*-glycoproteome. (A) Heatmap visualizing protein expression of cell type elevated (Tukey’s *p*-value < 0.05 and |log_2_FC| > 1 against two cell types) and shared (Tukey’s *p*-value < 0.05 and |log_2_FC| > 1 against one cell type) proteins detected in GLYCO experiments. (B) Statistically significant Gene Ontology: Biological Processes terms that were uniquely enriched in CAF-elevated proteins identified in the GLYCO fraction compared to WCL. Size of the circle represent the number of CAF-elevated *N*-glycoproteins that are annotated with the respective term and the shading of background indicates the adjusted *p*-value for the respective enrichment. (C) Bar plot showing cell line and tissue WCL detection [18] of 205 CAF elevated *N*-glycoproteins. The five CAF-elevated *N*-glycoproteins that were stromal enriched in tissue but not detected in WCL cell line samples are indicated. D) Hazard ratios for five CAF elevated *N*-glycoproteins that were stromal enriched in tissue but not detected in WCL in vitro samples. Bar plots show the Benjamini-Hochberg adjusted *p*-value from the log-rank test for the respective clinical outcome in the Zhang et al., cohort [20]. Table S1: Processed WCL and *N*-glycocapture data. Table S2: Statistically over-represented pathways in cell-type elevated and shared proteins. Table S3: Overall survival and recurrence-free survival statistics for the proteins in each cell type signature that were detected in the Zhang et al., 2016 dataset [20].
