# Supplementary material for: Proteomics of High-Grade Serous Ovarian Cancer Models Identifies Cancer-Associated Fibroblast Markers Associated with Clinical Outcomes
Source: Biomolecules. 2022 Dec 30;13(1):75. doi: 10.3390/biom13010075 (PMC9855416; doi:10.3390/biom13010075)
Supplement: Supplementary file 1 [file biomolecules-13-00075-s001.zip › BIOMOLECULES_SUPPLEMENTARY_figures.pdf]

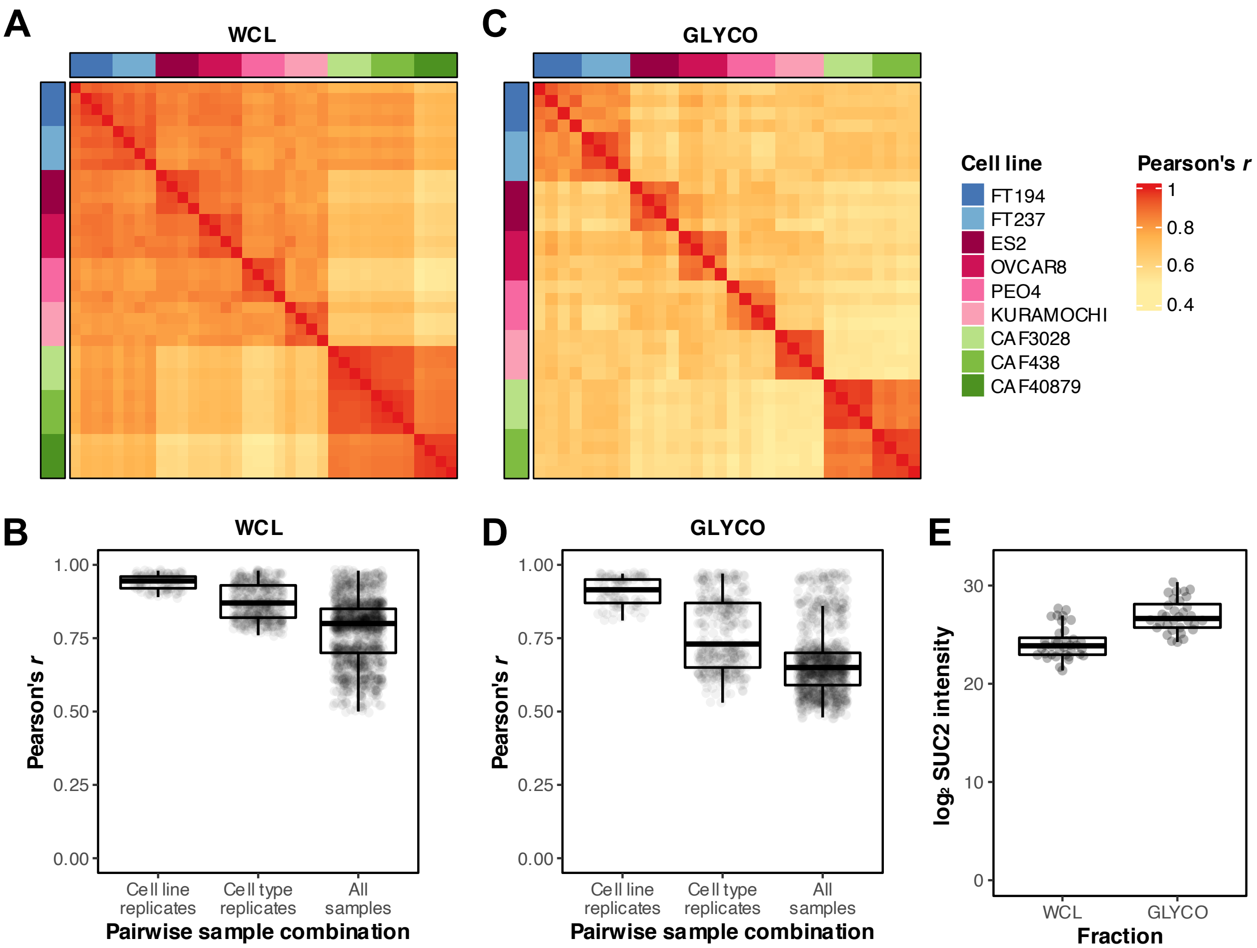

Figure S1: Quality control of proteomics data. A) Pearson correlation plot of protein intensities of all WCL samples. B) Box plot showing pairwise Pearson correlations between WCL cell line replicates (i.e., processing replicates, n=4), cell type replicates and all samples. Each dot represents a pairwise Pearson correlation. C) Pearson correlation plot of *N*-glycoprotein intensities of all GLYCO samples. D) Box plot showing pairwise Pearson correlations between GLYCO cell line replicates (i.e., processing replicates, n=4), cell type replicates and all samples. Each dot represents a pairwise Pearson correlation. E) Box plot of SUC2 protein intensity across all samples. Dots indicate SUC2 protein intensities in individual samples.

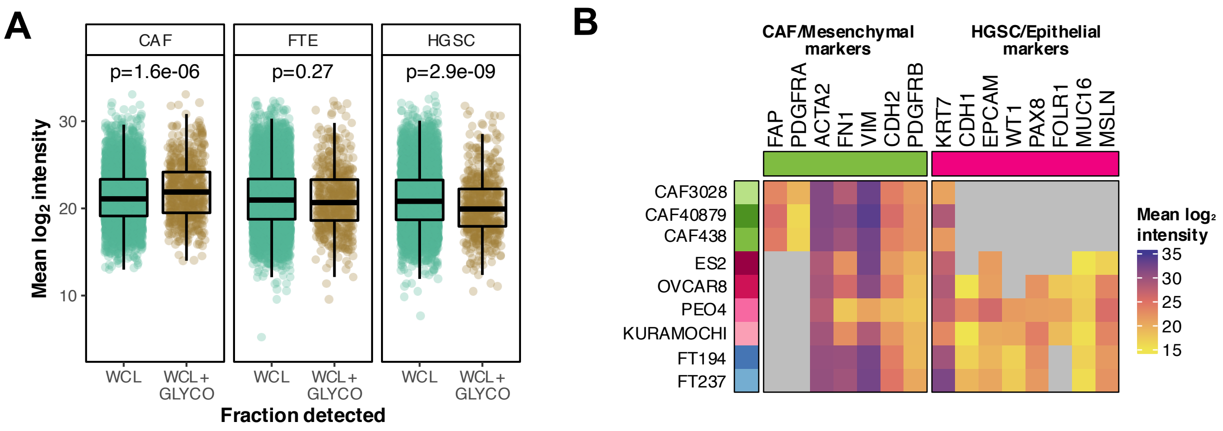

Figure S2: Additional proteomic cell type differences. A) Log_2intensity distributions of all proteins detected in WCL (green) and proteins detected in both WCL & GLYCO fractions (brown) for each cell type. Each dot represents a protein intensity. P-values from a Student's T-test are reported. B) Heatmap showing mean log_2protein intensity of known CAF/mesenchymal markers (green) and HGSC/epithelial markers (magenta) in WCL samples of all cell lines. Grey indicates that the protein was not detected in the cell line.

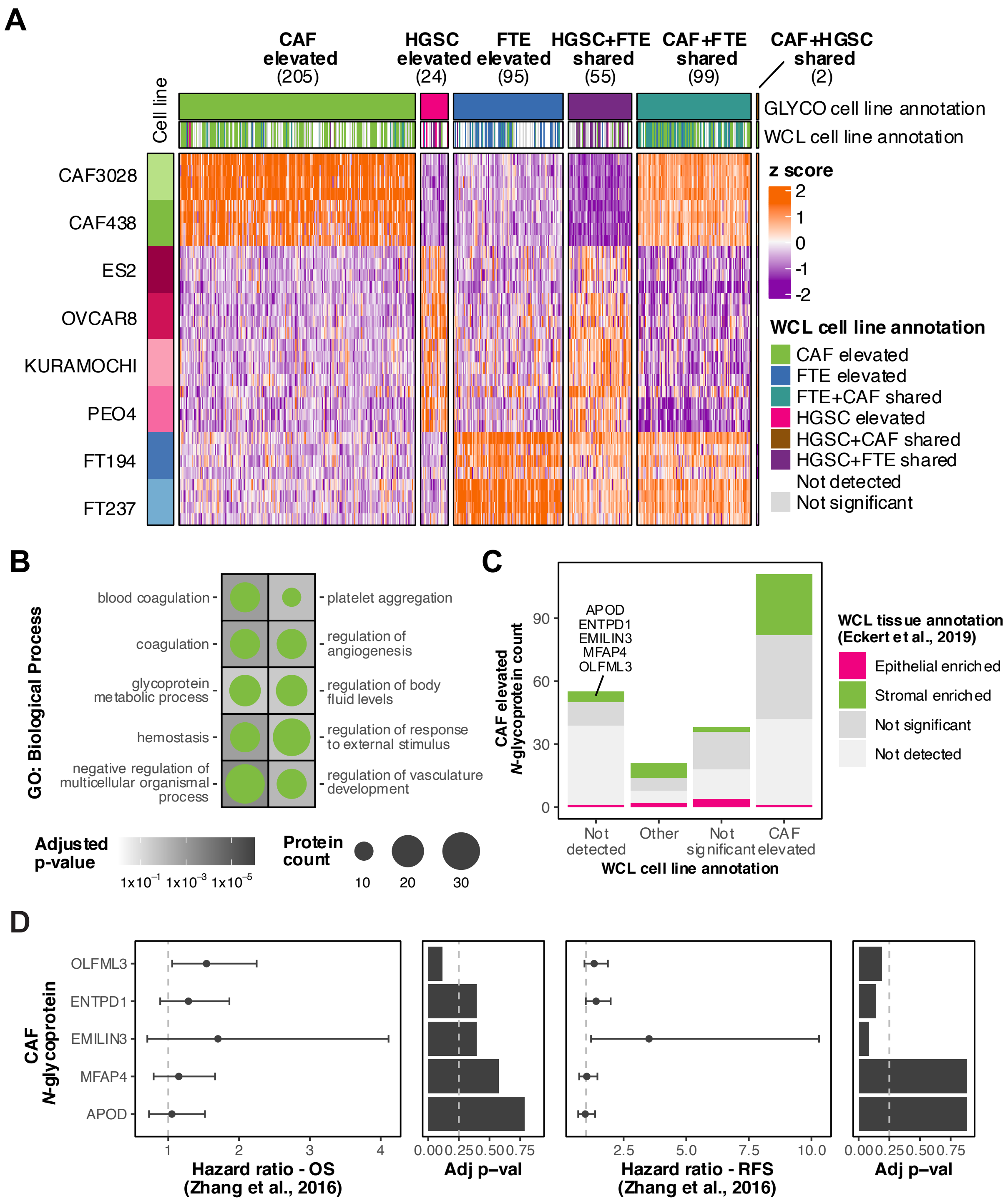

Figure S3: Characterization of the CAF elevated *N*-glycoproteome. A) Heatmap visualizing protein expression of cell type elevated (Tukey's p-value < 0.05 & $|log_2FC| > 1$ against two cell types) and shared (Tukey's p-value < 0.05 & $|log_2FC| > 1$ against one cell type) proteins detected in GLYCO experiments. B) Statistically significant Gene Ontology: Biological Processes terms that were uniquely enriched in CAF elevated proteins identified in the GLYCO fraction compared to WCL. Size of the circle represent the number of CAF elevated *N*-glycoproteins that are annotated with the respective term and the background shading indicates the adjusted p-value. C) Bar plot showing cell line and tissue WCL detection [18] of 205 CAF elevated *N*-glycoproteins. The five CAF elevated *N*-glycoproteins that were stromal enriched in tissue but not detected in WCL cell line samples are indicated. D) Hazard ratios for five CAF elevated *N*-glycoproteins that were stromal enriched in tissue but not detected in WCL *in vitro* samples. Bar plots show the Benjamini-Hochberg adjusted p-value from the log rank test for the respective clinical outcome in the Zhang et al., cohort [20].
